# Synthesis and Properties of New Dithienosilole Derivatives as Luminescent Materials

**DOI:** 10.3390/molecules24122259

**Published:** 2019-06-17

**Authors:** Dorota Zając, Damian Honisz, Mieczysław Łapkowski, Jadwiga Sołoducho

**Affiliations:** 1 Faculty of Chemistry, Wrocław University of Science and Technology, 50-370 Wrocław, Poland; dorota.zajac@pwr.edu.pl; 2 Faculty of Chemistry, Silesian University of Science and Technology, 44-100 Gliwice, Poland; damian.honisz@polsl.pl (D.H.); mieczyslaw.lapkowski@polsl.pl (M.L.); 3 Centre of Polymer and Carbon Materials of the Polish Academy of Sciences, 41-819 Zabrze, Poland

**Keywords:** dithienosilole, OLED, stille, Suzuki reaction, conjugated polymers, luminescence materials

## Abstract

Three new organosilicon compounds based on dithienosilole (DTSi) were synthesized in good yields. We report the optical and electrochemical properties of the resulting derivatives. We find that these compounds absorb the light in the ultraviolet and blue light range, and they exhibit luminescence in almost the entire range of visible light. After electropolymerization were significantly lowered, the values of the energy gap (even 1.51 eV for P2) and the ionization potential of the polymers were compared to monomers. Optoelectronic properties of the obtained compounds suggest that these derivatives of DTSi may be good candidates as the emissive layers in white organic light-emitting diodes (WOLEDs), which would reduce the amount of layers.

## 1. Introduction

In recent years, the design strategy of polymers and small molecule donor materials, including finely tuning conjugated polymer backbones, rationally changing the side chains, and suitably varying substituents, has received a great deal of attention [1]. Important compounds in these types of processes turn out to be silicon-containing aromatic compounds, e.g., dithienosilole (DTSi). Thanks to its unique properties, DTSi has often been used for π-conjugated functional materials [2,3,4]. They find application in the design of organic solar cells [5,6,7,8,9], organic light-emitting diodes [10,11,12,13,14,15], sensors [16,17,18,19], and organic field-effect transistors [20,21]. Silole has the lowest LUMO energy level and a relatively high HOMO level compared to many heteroarene monomer units of π-conjugated polymers, such as furan, thiophene, pyrrole, and pyridine [22]. DTSi compounds are stable, have good solubility in organic solvents, and can be handled without special requirements. However, their stability is better with larger substituents. It is worth mentioning here that the properties of DTSi are highly dependent on the substituents at the thiophene α-positions [23,24]. For example, DTSi derivatives with phosphine substituents have strong photoluminescent and electroluminescent properties, while the introduction of electron-donating methylthio groups gives the opportunity to use DTSi as hole-transporting materials for organic light emitting diodes [21]. The main purpose of this work is the synthesis of DTSi derivatives and the study of their optoelectronic properties, indicating the high potential of using these compounds in consumer electronics, e.g., in organic light-emitting diodes (OLED) diodes, biosensor, and organic solar cells.

## 2. Results and Discussion

### 2.1. Synthesis

**Scheme 1:**
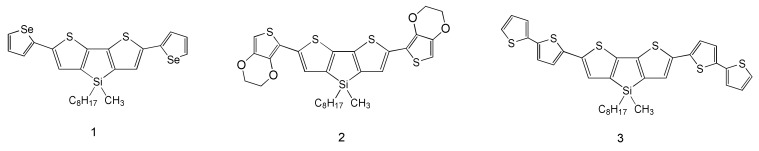
Chemical structure of **1**–**3** compound.

**Scheme 2:**
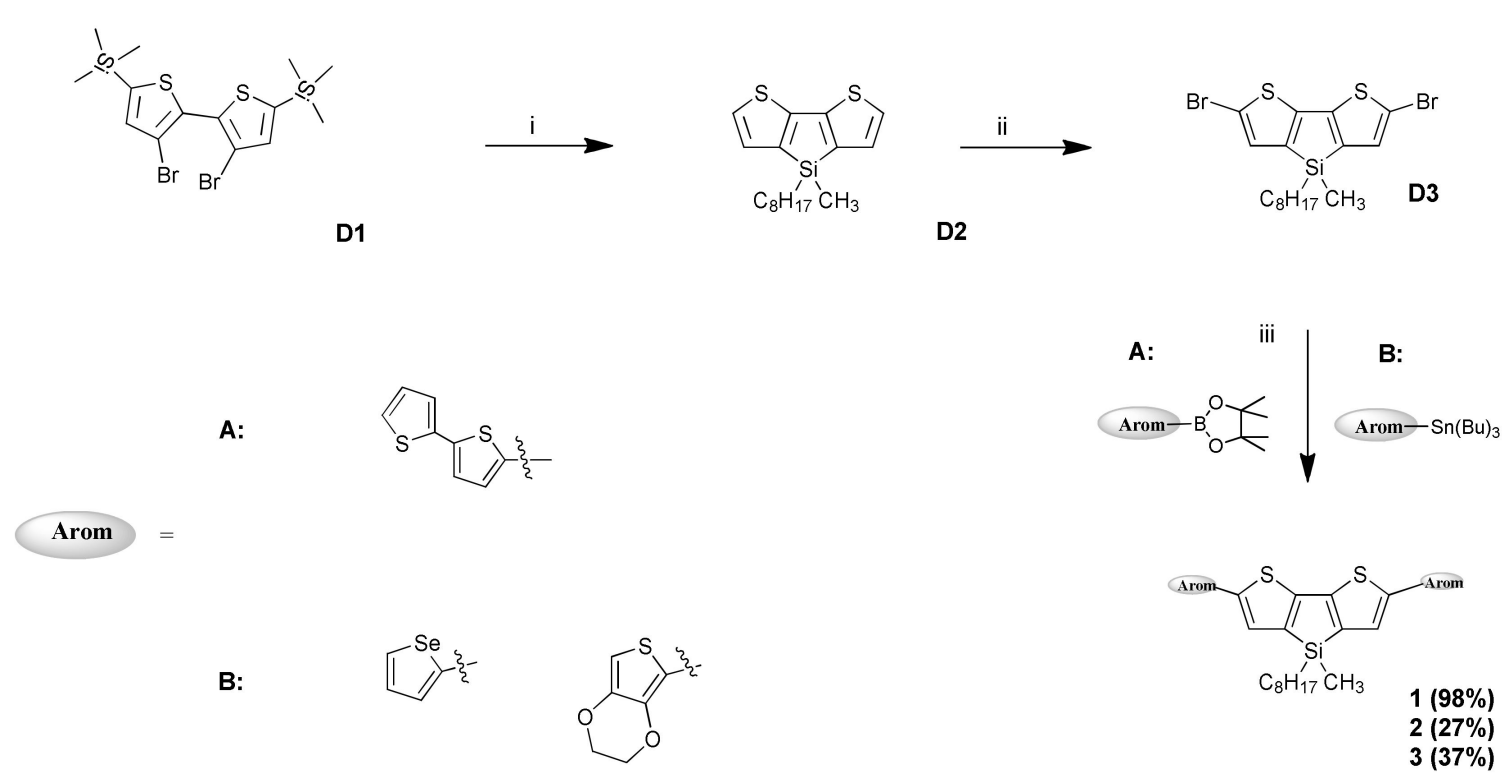
Synthesis of **1**–**3** compounds: (i): (**1**) n-BuLi, tetrahydrofurane (THF), N_2_, −78 °C, 1 h, (**2**) methyl-octyl-dichlorosilane; (ii): *N*-Bromosuccinimide (NBS),THF, 24 h; (iii): (**A**) K_2_CO_3_, Pd(PPh_3_)_4_, toluene, H_2_O, MeOH, N_2_, reflux, 24 h, (**B**) PdCl_2_(PPh_3_)_2_, THF, N_2_, reflux, 24 h.

The synthesized compounds **1–3** are shown in Scheme 1. The compound **D3** was obtained using our previously-described method [25]. The first step of the reaction was the synthesis of 4-methyl-4-octyl-dithienosilol (**D2**), which consisted of closing the thiophene ring by attachment in the β-β’-position of a silicon atom substituted with alkyl (methyl and *n*-octyl) groups. The introduction of a long aliphatic chain into the dithienosilole system improves the solubility of the compound and also affects the structural planarity, which is associated with the improvement of conductive properties. The final product was obtained by Suzuki coupling for compound **3**, while compounds **1** and **2** were obtained using the Stille reaction method. These reactions occurred with a very different yield of reaction (from 27% to 98%). The structures of the compounds were characterized by ^1^H-NMR, ^13^C-NMR; MS spectra measurements could not be performed since these compounds were disintegrated before reaching the detector. The synthesis of compounds **1–3** is shown in Scheme 2.

### 2.2. Optical Properties

After the compounds were identified, the optical properties of absorption and fluorescence were measured. The standard test is dichloromethane. The test results, both for the measurement of fluorescence and absorbance, was 10^−6^ mol/dm^3^.

**Figure 1:**
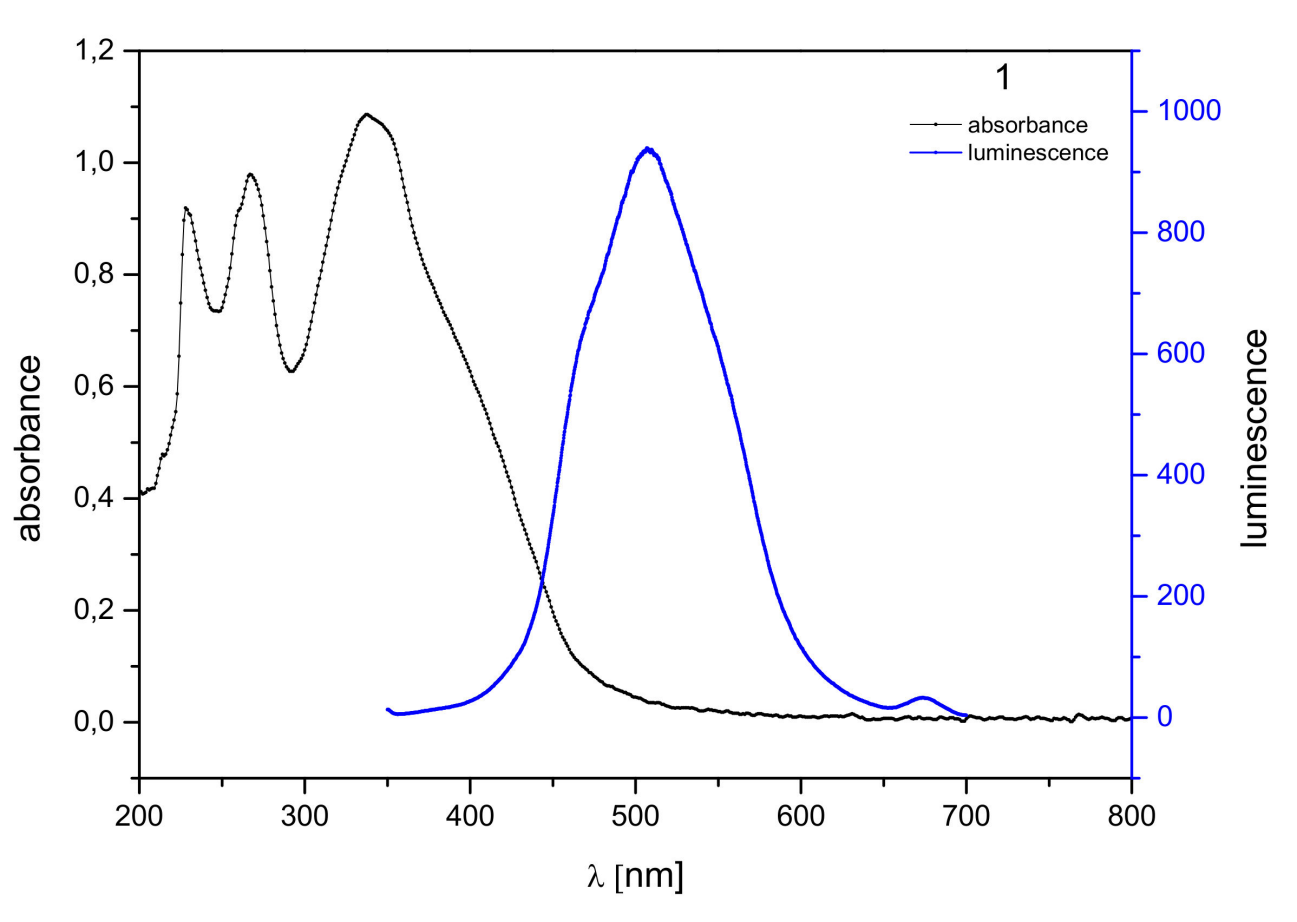
Normalized absorption spectra (black) and emission spectra (blue) of **1** in dichloromethane solution (10^−6^ mol/dm^3^).

The maximum absorption of **1** is at the wavelength λ_max_ = 338 nm (Figure 1). During the spontaneous transition of electrons from a higher energy level to a lower one, as a result of absorbing electromagnetic radiation, we observed fluorescence in the visible light region, ranging from 410 nm to 620 nm, with the maximum at λ_max_ = 506 nm. The Stokes shift was 168 nm.

**Figure 2:**
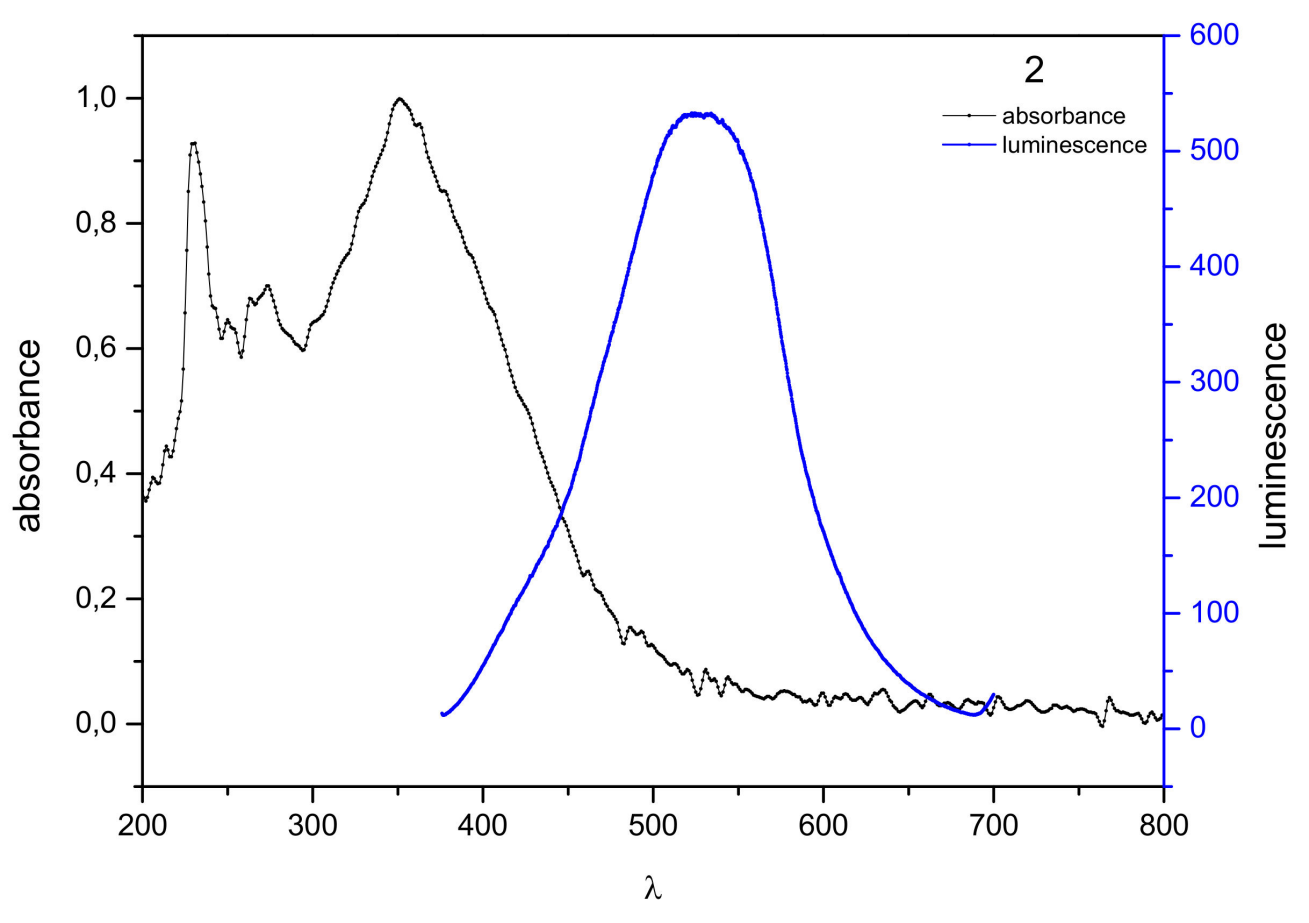
Normalized absorption spectra (black) and emission spectra (blue) of **2** in dichloromethane solution (10^−6^ mol/dm^3^).

The maximum absorption of **2** is at the wavelength λ_max_ = 351 nm (Figure 2). The absorption band shows the absorption of the compound in the ultraviolet and blue visible light (from 300 to about 500 nm). The observed emission is in the area of near ultraviolet and visible light, from 380 nm to 640 nm, with excitation at 530 nm. The Stokes shift was 209 nm. The large Stokes shift values suggest that the obtained derivatives **1–3** are very interesting materials for use in optoelectronics and sensor devices, and also may find application as effective fluorophores with an ordered structure, improving the sensitivity of detection with a lower frequency of fluorescence quenching [26].

**Figure 3:**
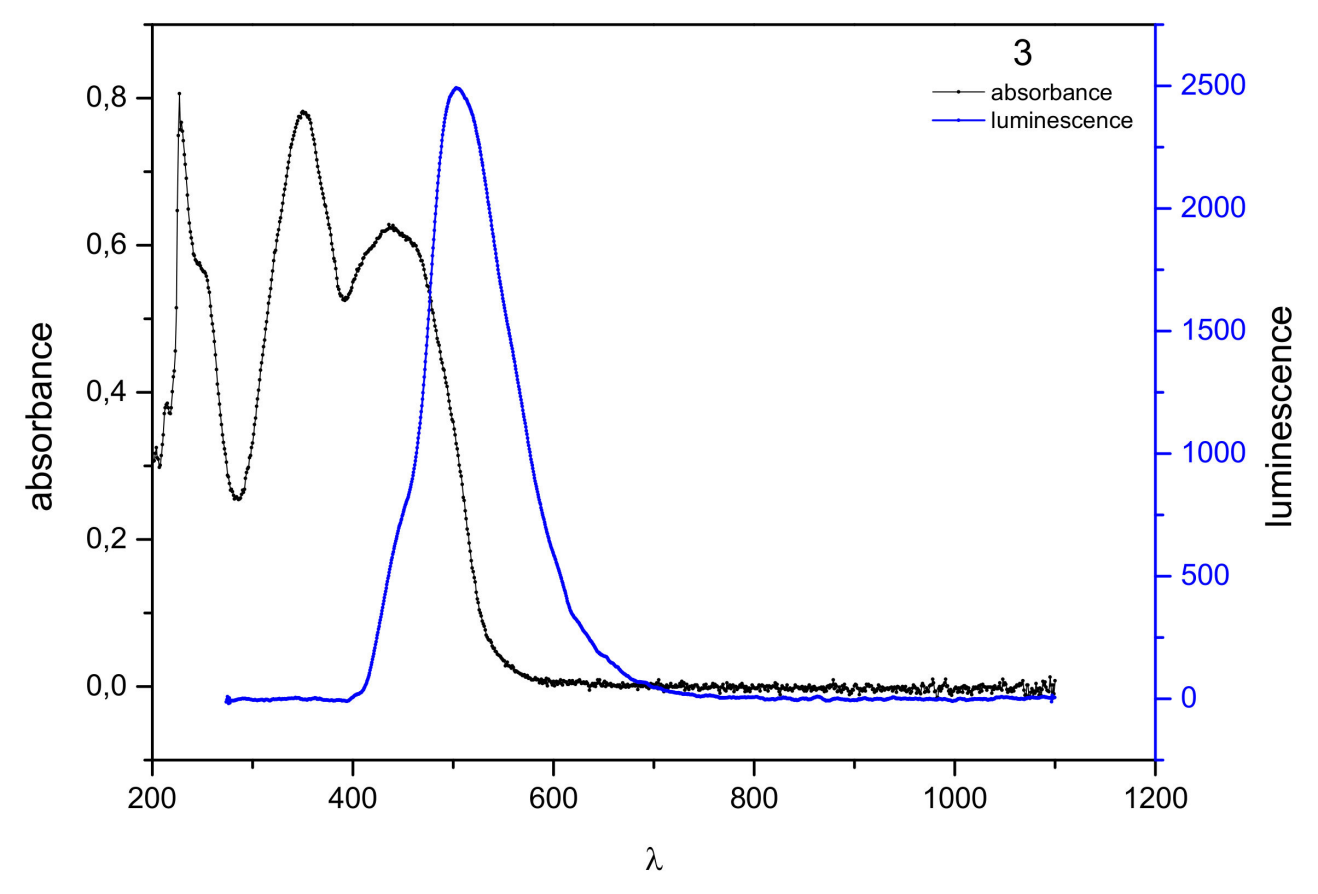
Normalized absorption spectra (black) and emission spectra (blue) of **3** in dichloromethane solution (10^−6^ mol/dm^3^).

The maximum absorption of **3** is at the wavelength λ_max_ = 350 and 436 nm (Figure 3). The absorption band, corresponding to the π–π * transition, was observed in the range from 300 to about 520 nm. The observed fluorescence is in the area of near ultraviolet and visible light, from 400 nm to 670 nm, with excitation at 503 nm. The Stokes shift was 153 and 67 nm.

### 2.3. Electrochemical Properties

**Figure 4:**
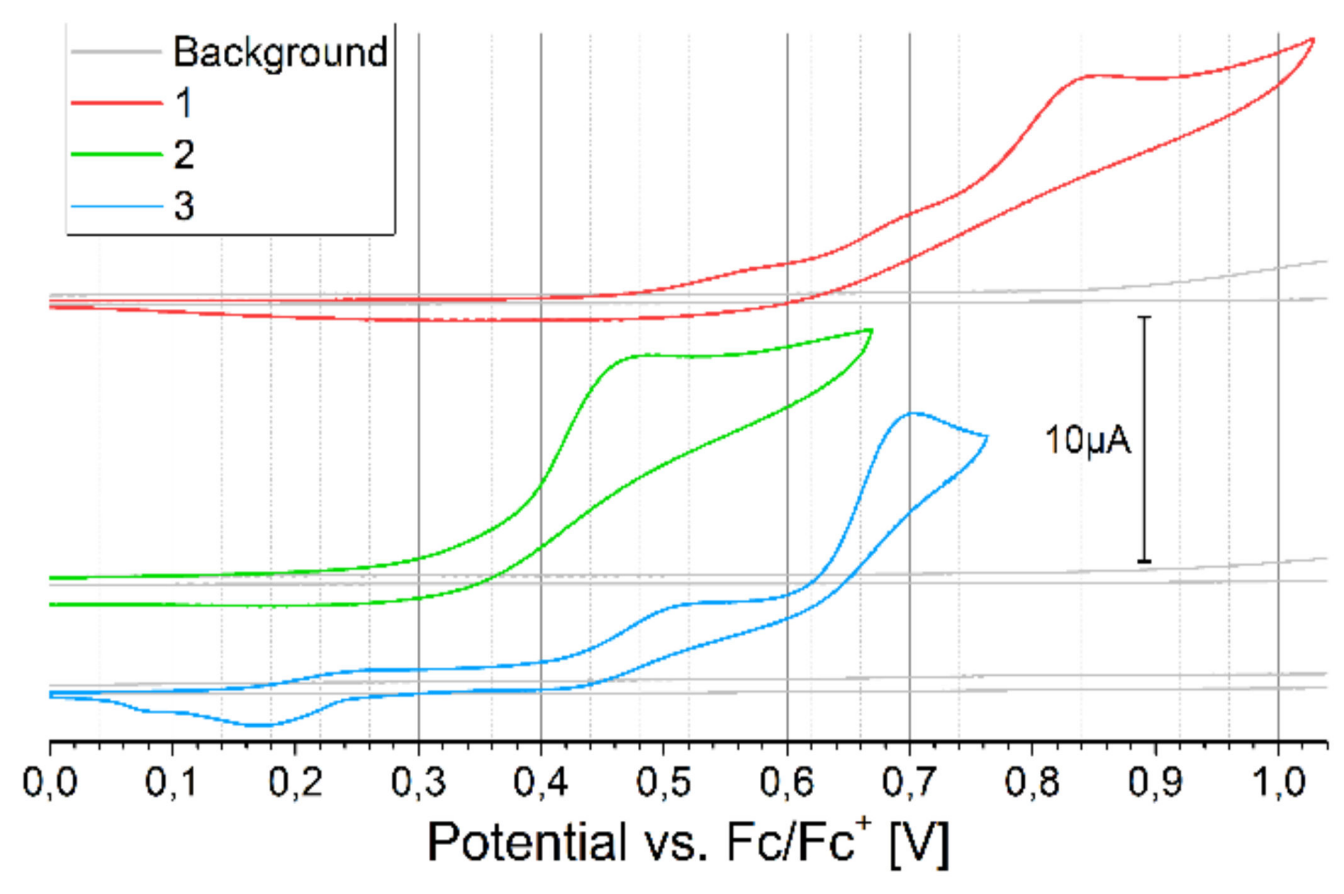
The cyclic voltammetry (CV) of oxidation of monomers versus ferrocene redox couple, concentration 2 mmol/dm^3^ in DCM, 100 mV/s.

**Figure 5:**
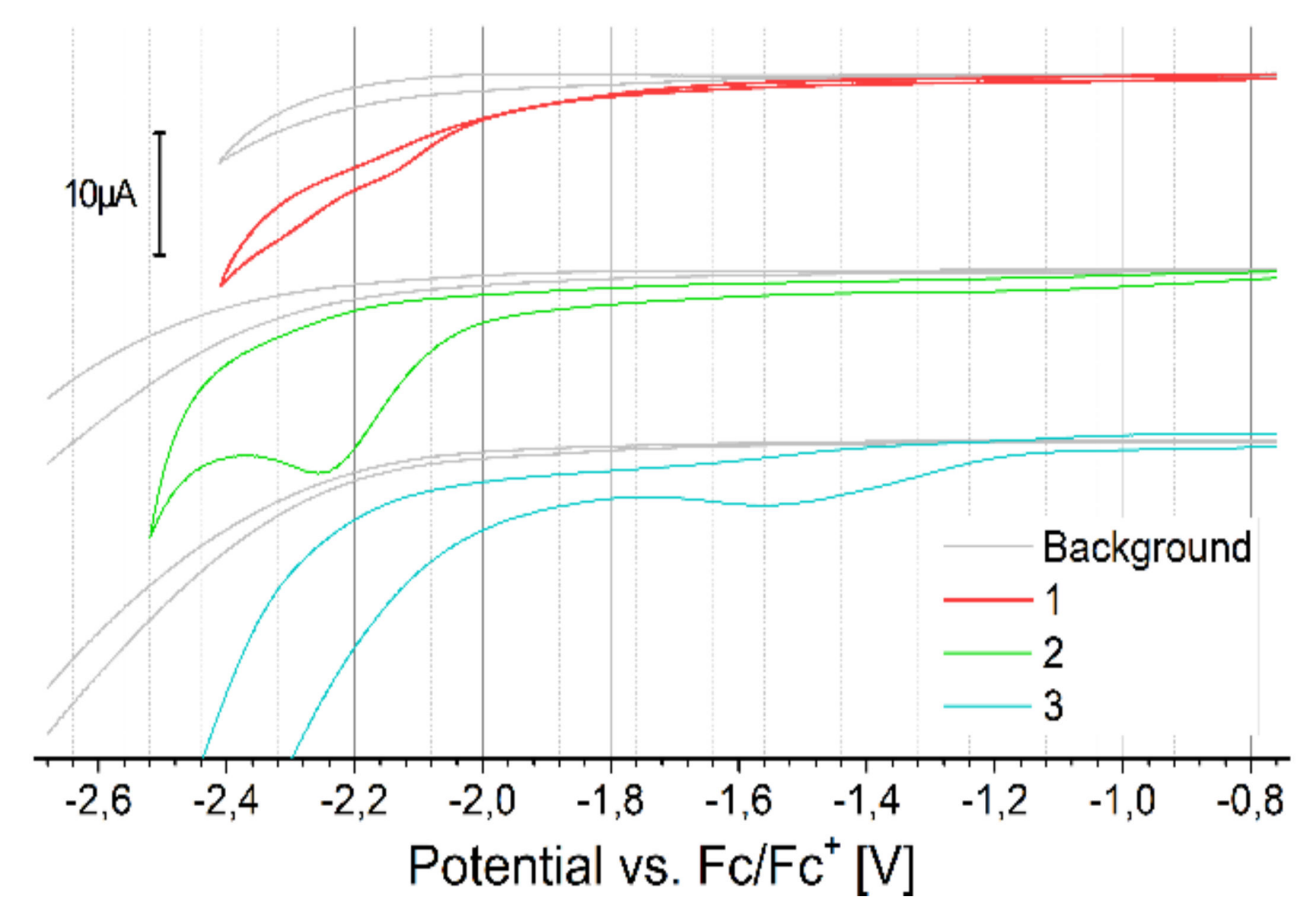
The CV of reduction of monomers versus ferrocene redox couple, concentration 2 mmol/dm^3^ in dichlorometane (DCM), 100 mV/s.

Cyclic voltammetry (CV) of monomers oxidation versus the ferrocene redox couple, with the background measurements of a pure electrolyte solution, is presented in Figure 4. All monomers oxidize irreversibly in multi-step process. Before the first oxidation peak of compound 3 (0.42 V onset), one can see a small peak with onset 0.15 V, a representation of short oligomer contamination formed in spontaneous oxidation of monomer on air. CV of the reduction process is presented in Figure 5.

The monomers show similar reduction potential, again, for compound **3**, the oligomer peak is visible at a potential higher than for the monomer. All electrochemical results for the monomers are summarized in Table 1. Comparing the oxidation peak, ionization potential, and energy gap of analogous structures/polymers available in the literature, it can be concluded that for derivative **1**, the chain length and branching affects the closer packing of the compound (increasing the E_ox_ value (0.34 V vs. Fc/Fc^+^ (potential converted according to [27]) for the bis(C_8_H_17_) chain, 0.48V in the case of compound **1** and IP))[28]. By adding an additional thiophene group as a donor moiety in compound **3**, only slightly increases the E_ox_ and IP values by increasing the elongation of the conjugated unit, which abolish effect of changing the aliphatic chain from symmetric bis(C_8_H_17_) to methyl-octyl [28]. In the case of derivative **2**, to our knowledge, there is no sufficiently similar structure to compare.

**Figure 6:**
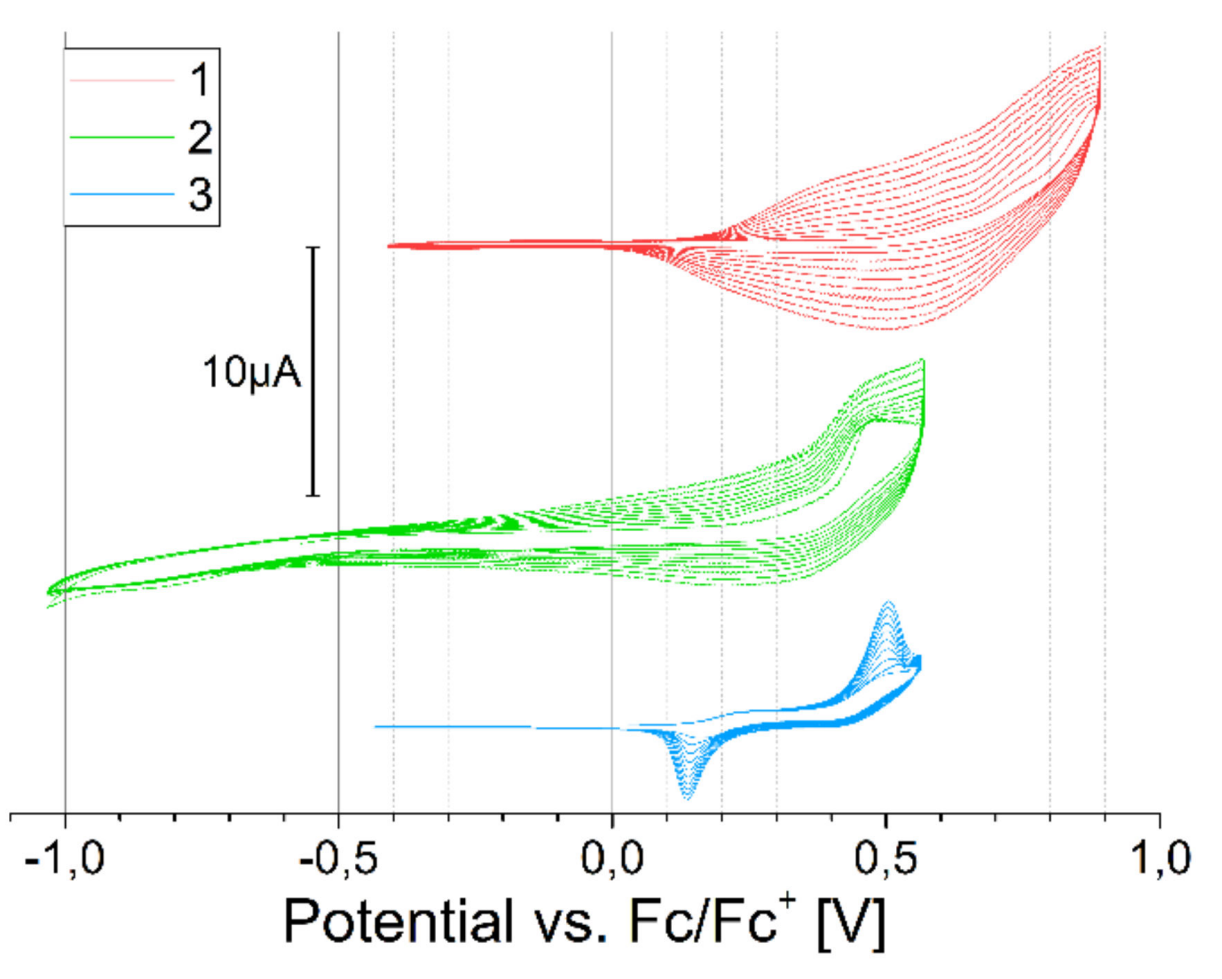
Cyclic polymerization versus ferrocene redox couple, concentration 2 mmol/dm^3^ in DCM, 100 mV/s.

All compounds polymerize well (Figure 6); however, the sharp polymerization peak of **3** indicates a short conjugation length in the product. This is likely caused by polymer chain defects, a coupling reaction involving the β-position on thiophene group, which is more likely for bithiophene substituents [29].

**Figure 7:**
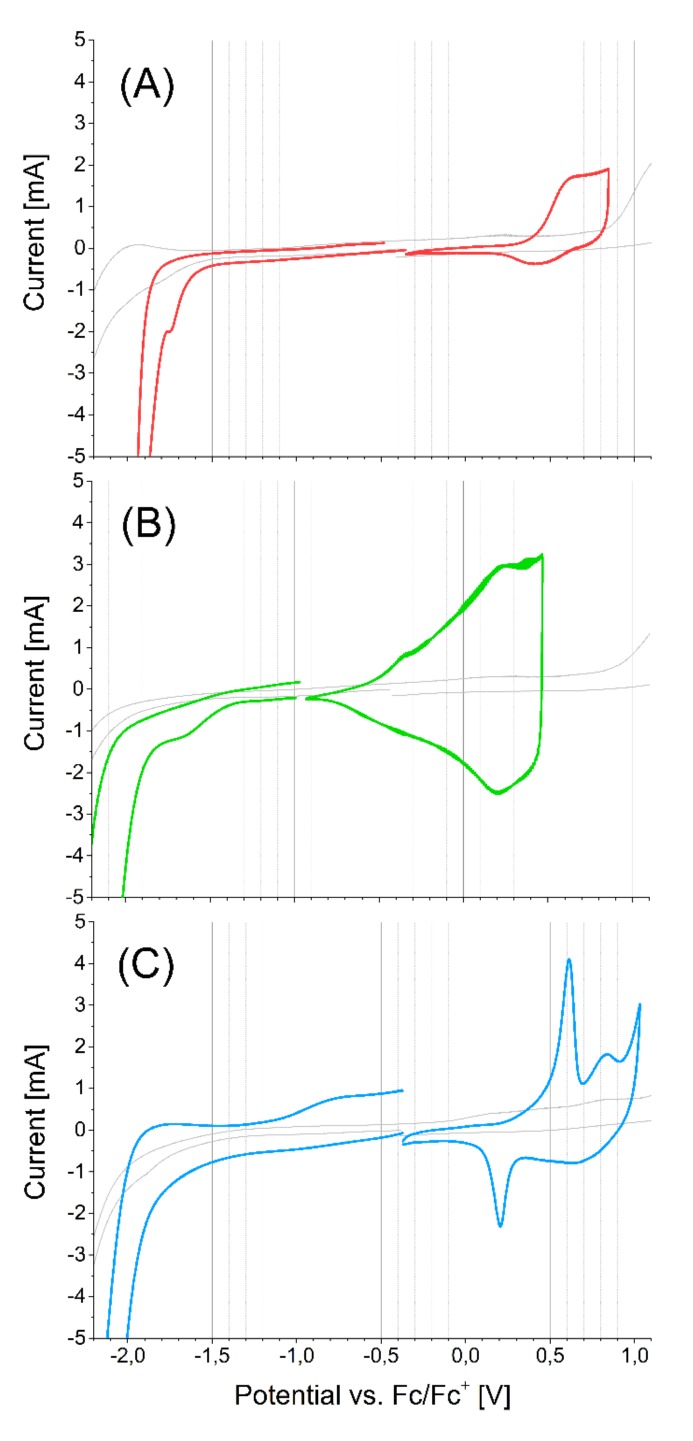
The CV of (**A**)-P1, (**B**)-P2, (**C**)-P3 polymers films on Pt wire versus ferrocene redox couple in DCM, 100 mV/s.

The CVs of polymers are shown in the Figure 7. The electrochemical band gap of the polymer P2 is very low, such that the ionization potential (IP) of P2 is shown as much lower than its monomer. It is well known for EDOT containing polymers, because of sulfur–oxygen interaction between adjacent EDOT unit, that enforces planarity and increases conjugation. Electrochemical properties of electropolymerized polymers are summarized in Table 2.

## 3. Materials and Methods

### 3.1. Chemistry

*n*-Butyllithium (2.5 M in hexane), 3,4-ethylenedioxythiophene (97%), bis(triphenylphosphine)palladium (II) dichloride (98%) were purchased from Aldrich. Selenophene (98.0%) and 5-(4,4,5,5-tetramrthyl-1,3,2-dioxaborolan-2-yl-2,2′-bithiophene (98%), were purchased from TCI Europe. Anhydrous potassium carbonate (99%) was received from Chempur. Anhydrous tetrahydrofuran was purchased from POCH. Tetrahydrofuran was dried over Na/benzophenone ketal before use. Other commercially available substances and reagents were used without the prior purification. Preparative column chromatography was performed on the glass column with Acros Organics silica gel for chromatography, 0.035–0.075 mm; 60 Å. The 600 MHz ^1^H NMR and ^13^C NMR spectra were recorded in deuterated chloroform (CDCl_3_) on Bruker Avance II 600 Instruments, respectively. Chemical shifts were locked to chloroform δ_H_ 7.26 (s) and δ_C_ 77.16 (t) signals.

#### 3.1.1. Preparation of 2,6-Di(selenophen-2-yl)-4-Methyl-4-Octyl-Dithienosilole (**1**)

To a mixture of **D3** (1.24 g, 2.50 mmol) and 2-(tributylstannyl)selenophene (2.39 g, 5.68 mmol) in anhydrous THF (60 mL) was added bis(triphenylphosphine)palladium (II) dichloride (PdCl_2_(PPh_3_)_2_) (0.351 g, 0.520 mmol) at room temperature under nitrogen atmosphere. The resulting mixture was refluxed with stirring for 24 h. Then, the reaction mixture was concentrated under reduced pressure, diluted with water, and extracted with EtOAc. The extract was washed with brine, dried over MgSO_4_, and concentrated. The residue was purified by silica gel column chromatography (hexane-EtOAc in the gradient of polarity) to give **1** (1.47 g, 98.0%) as a red oil.

- ^1^H NMR (600 MHz, CDCl_3_) δ (ppm): δ 7.84 (d, *J* = 5.6 Hz, 2H), 7.59 (d, *J* = 7.6 Hz, 2H), 7.28 (d, *J* = 3.6 Hz, 2H), 7.08 (s, 2H), 1.24–1.23 (m, 12H), 0.93–0.89 (m, 2H), 0.85 (t, *J* = 6.8 Hz, 3H), 0.41 (s, 3H).
- ^13^C NMR (600 MHz, CDCl_3_) δ (ppm): δ 143.5, 140.8, 140.6, 138.7, 135.1, 132.5, 131.0, 130.8, 130.4, 129.1, 129.0, 128.6, 127.3, 127.1, 126.7, 125.7, 125.4, 33.2, 31.9, 29.8, 29.3, 24.2, 22.7, 14.3, 13.3.

#### 3.1.2. Preparation of 2,6-Bis(3,4-Ethylenedioxythiophen-2-yl)-4-Methyl-4-Octyl-Dithienosilole (**2**)

This compound was prepared by using the same procedure as was used for **1**. To a mixture of **D3** (1.00 g, 2.10 mmol) and 2-(tributylstannyl)ethylenedioxythiophene (1.98 g, 4.60 mmol) in anhydrous THF (50 mL) was added bis(triphenylphosphine)palladium (II) dichloride (PdCl_2_(PPh_3_)_2_) (0.295 g, 0.420 mmol) at room temperature under nitrogen atmosphere. The resulting mixture was refluxed with stirring for 48 h. Then, the reaction mixture was concentrated under reduced pressure, diluted with water, and extracted with EtOAc. The extract was washed with brine, dried over MgSO_4_, and concentrated. The residue was purified by silica gel column chromatography (hexane-EtOAc in the gradient of polarity) to give **2** (0.338 g, 27.0%) as a dark green oil.

- ^1^H NMR (400 MHz, CDCl_3_)δ (ppm): δ 7.16 (s, 2H), 6.27 (s, 2H), 4.25–4.33 (m, 4H), 4.25–4.23 (m, 4H), 1.24–1.17 (m, 12H), 0.96–0.93 (m, 2H), 0.84 (t, *J* = 10.2 Hz, 3H), 0.63 (s, 3H).
- ^13^C NMR (600 MHz, CDCl_3_)δ (ppm): δ 141.90, 138.76, 136.72, 126.41, 125.65, 112.38, 110.65, 98.40, 65.19, 64.62, 33.56, 32.03, 29.78, 29.39, 26.97, 26.65, 26.38, 22.78, 13.58.

#### 3.1.3. Preparation of 2,6-Di([2,2’-Bithiophen]-5-yl)-4-Methyl-4-Octyl-Dithienosilole (**3**)

To a mixture of **D3** (1.00 g, 2.10 mmol), potassium carbonate (0.578 g, 4.18 mmol), and 5-(4,4,5,5-tetramethyl-1,3,2-dioxaborolan-2-yl-2,2′-bithiophene (1.83 g, 6.27 mmol) in toluene (45 mL), MeOH (10 mL), and water (10 mL) was added bis(triphenylphosphine)palladium (II) dichloride (PdCl_2_(PPh_3_)_2_) (0.293 g, 0.418 mmol) at room temperature. The resulting mixture was refluxed with stirring for 24 h under nitrogen atmosphere. Then, the reaction mixture was concentrated under reduced pressure, diluted with water, and extracted with EtOAc. The extract was washed with brine, dried over MgSO4, and concentrated. The residue was purified by silica gel column chromatography (hexane-EtOAc in the gradient of polarity) to give **3** (0.50 g, 37.0%) as an orange oil.

- ^1^H NMR (600 MHz, CDCl_3_)δ (ppm): δ 7.64 (d, *J* = 8.4 Hz, 2H), 7.42 (t, *J* = 7.8 Hz, 2H), 7.27–7.21 (m, 4H), 7.18 (d, *J* = 3.6 Hz, 1H), 7.11 (d, *J* = 3.0 Hz, 1H), 7.07–7.04 (m, 2H), 1.39–1.27 (m, 12H), 1.08 (t, *J* = 7.8 Hz 2H), 0.92 (t, *J* = 7.2 Hz, 3H), 0.47 (s, 3H).
- ^13^C NMR (600 MHz, CDCl_3_) δ (ppm): δ 143.76, 142.14, 138.16, 137.30, 136.60, 136.44, 135.88, 134.16, 129.04, 128.01, 127.68, 126.32, 125.71, 124.70, 124.50, 124.47, 123.96, 123.81, 123.72, 123.69, 33.23, 32.00, 29.30, 29.20, 27.78, 27.50, 24.23, 22.80, 14.24, 13.80, 13.30.

### 3.2. Cyclic Voltammetry

For the cyclic voltammetry (CV) experiments, a three-electrode glass cell was used with a platinum wire as a working electrode, a platinum wire spiral as a counter electrode, and an Ag/Ag^+^ reference using 0.1 M Bu_4_NPF_6_ (TCI Europe, Zwijndrecht, Belgium) electrolyte solution in dichloromethane (DCM) (Chromasolv, HPLC; Sigma-Aldrich, St. Louis, MI, USA). The potential sweeps were controlled by Metrohm Autolab PGSTAT 100N potentiostat (Metrohm Autolab B.V., Utrecht, The Netherlands). The potential of the silver electrode was determined using a ferrocene redox couple (Fc/Fc^+^) for each measurement set, under the same conditions as the measured samples. The solutions were deaerated with argon before, and argon kept flowing into the cell, above the solution surface, during measurements. Two mmol/dm^3^ concentrations of the monomers were used for both the measurements and polymerization.

## 4. Conclusions

A series of dithienosilole derivatives were obtained in good yield. In the case of the selenophene derivative, it was obtained up to 98% yield, which gives an excellent result in Stille reaction. All compounds absorb light in the ultraviolet and blue range, while they exhibit luminescence in almost the entire range of visible light. This fact, and large values of Stokes shift, make these compounds very promising in terms of their potential use in optoelectronics, especially in WOLEDs. In addition, these compounds were electropolymerized and the resulting films exhibit very narrow energy gaps (0.75 eV for P2, 1.52 eV for P3 and 1.75 for P1) and low ionization potentials (4.47–5.37 eV) compared to monomers (Eg in the range 2.23–2.29 eV, while IP is in the range 5.49–5.58 eV). This allows efficient control of the film’s parameters to obtain a layer with very good electroconductive properties.

## Figures and Tables

**Table 1:** Redox properties of monomers. IP—ionization potential, estimated from equation IP [eV] = |*e*|5.1 + E^ox^_onset_ [V]; EA—electron affinity, estimated from equation EA [eV] = |*e*|5.1 + E^red^_onset_ [V]; ΔE_g_^el^ —electrochemical bandgap from equation ΔE_g_^el^ = IP−EA; 3a—dimer contamination in sample 3.

Compound	E^ox^_onset_ (V)	E^red^_onset_ (V)	IP (eV)	EA (eV)	ΔE_g_^el^ (eV)
1	0.48	−1.81	5.58	3.29	2.29
2	0.39	−1.87	5.49	3.23	2.26
3	0.42	−1.81	5.52	3.29	2.23
3a	0.15	−1.18	5.25	3.92	1.33

**Table 2:** Redox properties of polymers. IP—ionization potential, estimated from equation IP [eV] = |*e*|5.1 + E^ox^_onset_ [V]; EA—electron affinity, estimated from equation EA [eV] = |*e*|5.1 + E^red^_onset_ [V]; ΔE_g_^el^ —electrochemical bandgap, from equation ΔE_g_^el^ = IP−EA.

Compound	E^ox^_onset_ (V)	E^red^_onset_ (V)	IP (eV)	EA (eV)	ΔE_g_^el^ (eV)
P1	0.27	−1.48	5.37	3.62	1.75
P2	−0.63	−1.34	4.47	3.76	0.71
P3	0.21	−1.31	5.31	3.79	1.52
